# PD-1 and PD-L1 Expression in NSCLC Indicate a Favorable Prognosis in Defined Subgroups

**DOI:** 10.1371/journal.pone.0136023

**Published:** 2015-08-27

**Authors:** Lars Henning Schmidt, Andreas Kümmel, Dennis Görlich, Michael Mohr, Sebastian Bröckling, Jan Henrik Mikesch, Inga Grünewald, Alessandro Marra, Anne M. Schultheis, Eva Wardelmann, Carsten Müller-Tidow, Tilmann Spieker, Christoph Schliemann, Wolfgang E. Berdel, Rainer Wiewrodt, Wolfgang Hartmann

**Affiliations:** 1 Department of Medicine A, Hematology, Oncology and Pneumology, University Hospital Muenster, 48149 Muenster, Germany; 2 Pulmonary Division, Department of Medicine III, Johannes Gutenberg University Medical Center, 55101 Mainz, Germany; 3 Institute of Biostatistics and Clinical Research, University of Münster, Münster, Germany; 4 Gerhard-Domagk-Institute of Pathology, University of Münster, Münster, Germany; 5 Chest Surgery, Klinikum Bremen Ost, 28325 Bremen, Bremen, Germany; 6 Department of Pathology, Memorial Sloan Kettering Cancer Center, New York, United States of America; 7 Department of Medicine IV, Hematology and Oncology, University of Halle, 06120 Halle, Germany; 8 Institute for Pathology at St. Franziskus-Hospital, Münster, 48145 Münster, Germany; University of Barcelona, SPAIN

## Abstract

**Background:**

Immunotherapy can become a crucial therapeutic option to improve prognosis for lung cancer patients. First clinical trials with therapies targeting the programmed cell death receptor PD-1 and its ligand PD-L1 have shown promising results in several solid tumors. However, in lung cancer the diagnostic, prognostic and predictive value of these immunologic factors remains unclear.

**Method:**

The impact of both factors was evaluated in a study collective of 321 clinically well-annotated patients with non-small lung cancer (NSCLC) using immunohistochemistry.

**Results:**

PD-1 expression by tumor infiltrating lymphocytes (TILs) was found in 22%, whereas tumor cell associated PD-L1 expression was observed in 24% of the NSCLC tumors. In Fisher’s exact test a positive correlation was found for PD-L1 and Bcl-xl protein expression (p = 0.013). Interestingly, PD-L1 expression on tumor cells was associated with improved overall survival in pulmonary squamous cell carcinomas (SCC, p = 0.042, log rank test), with adjuvant therapy (p = 0.017), with increased tumor size (pT2-4, p = 0.039) and with positive lymph node status (pN1-3, p = 0.010). These observations were confirmed by multivariate cox regression models.

**Conclusion:**

One major finding of our study is the identification of a prognostic implication of PD-L1 in subsets of NSCLC patients with pulmonary SCC, with increased tumor size, with a positive lymph node status and NSCLC patients who received adjuvant therapies. This study provides first data for immune-context related risk stratification of NSCLC patients. Further studies are necessary both to confirm this observation and to evaluate the predictive value of PD-1 and PD-L1 in NSCLC in the context of PD-1 inhibition.

## Introduction

Lung cancer remains one of the most common and one of the most lethal cancers worldwide [1]. Throughout the last decade distinct molecular factors were identified as driving tumor growth and spread and/or as being prognostic in non-small cell lung cancer (NSCLC). Several attempts followed to specifically target these factors and thereby influence the clinical course of disease. Molecular based therapies targeting epidermal growth factor receptor (EGFR) mutants [2,3] or ALK rearrangements [4] were shown to improve the outcome within well-defined subgroups of non-squamous cell carcinoma patients. So far, only for a minority of all patients, targetable genetic alterations have been identified. NSCLC patients with progressive disease and without targetable alterations are treated with traditional chemotherapies. The majority of them suffers from chemotherapy-associated toxicities and poor overall survival due to chemotherapy resistance. The recognition of cancer by the immune system and mechanisms of cancer to escape the immune control are areas of increasing research interest. To enlarge our therapeutic armamentarium, new potent antigens need to be identified. In the future, novel and modified immunotherapeutic concepts might improve cancer cell recognition for effective tumor control.

Although an increased CD4^+^/CD8^+^ cell infiltration of the tumor stroma has previously been shown to represent a favorable prognostic factor in NSCLC [5], ineffective therapeutic approaches with IL-2 [6] and interferon [7] have led to the conclusion that NSCLC is non-immunogenic. Recent gains of information in the field of tumor immunology include identification of key regulators of immune responses with broad impact on natural and therapeutic antitumor immune responses. The best-characterized immunological checkpoints with a major impact on both cancer growth and cancer therapy are cytotoxic T-lymphocyte antigen 4 (CTLA-4) and programmed death receptor 1 (PD-1) and their respective ligands. Both receptors (i.e. CTLA-4 and PD-1) inhibit T cell activation through distinct and potentially synergistic mechanisms. While CTLA-4 fails to downregulate the survival gene Bcl-xl, PD-1 engagement is suggested to induce T cell apoptosis [8]. The PD-1 receptor is expressed by CD4^+^ and CD8^+^ lymphocytes, regulatory T cells (Tregs) and B lymphocytes [9]. Upregulation of PD-1 modulates peritumoral inflammatory processes [10]. Consequently, binding of PD-1 by the two major ligands PD-L1 (CD274) or PD-L2 (CD 273) inhibits cytokine production. Inflammatory cytokines are reported to induce PD-L1 expression in tumor cells. PD-L1 interacts with PD-1 on T cells and downregulates T cell effector functions. This mechanism can enable cancer cells to evade host immune surveillance. Indeed, in several tumor types, increased PD-1 levels were found in tumor infiltrating lymphocytes (TILs) [11]. Besides adaptive PD-L1 upregulation in an inflammatory cytokine milieu, tumors can have innate potential to inactivate PD-1 by oncogene driven PD-L1 tumor expression [12]. Antibodies against the PD-1-pathway have been successfully applied to reverse T cell tolerance of malignant cells and induce tumor regression [12]. In first clinical studies, anti-PD-1 antibodies have shown activity in some NSCLC patients [13]. While these antibodies are undergoing clinical evaluation in lung cancer and other malignancies, the concrete biological significance of PD-1 and PD-L1 expression in cancer remains unclear. PD-L1-positivity was found to be associated with an inflammatory tumor microenvironment in lung cancers with squamous cell carcinoma [14] or adenocarcinoma histology [15]. Recently, one study revealed high PD-L1 expression by tumor cells to predict complete response as evaluated by histopathology to pre-operative chemotherapy in breast cancer [16]. In the context of the emerging PD-1 pathway inhibitors, the particular characteristics of patients with expected therapeutic response to these agents still need to be defined.

The present study aims at the evaluation of the prognostic significance of PD-1 expression in TILs and PD-L1 expression in NSCLC tumor cells in a large study collective of NSCLC using immunohistochemistry, with a particular emphasis on clinicopathological parameters.

## Methods

### Study population

Clinical follow up information and sufficient tumor material of 321 curatively resected NSCLC patients (median age: 66 years) from the Thoracic Departments in Ostercappeln, Germany (study collective I; n = 265 NSCLC tissue samples) and Mainz, Germany (study collective II; n = 56 NSCLC tissue samples) were collected. Approval of the study by the Ethical committee of Münster and Mainz were obtained for the collection of paraffin embedded tissue samples for biomarker testing. Due to the retrospective, anonymized character of the analysis, written consent was not required. Clinical TNM staging (including clinical examination, CT scans, sonography, endoscopy, MRI, bone scan) was based on IUCC/AJCC recommendations. Patients with stage IV, R1 or R2 resection status or with non-specified tumor histology (e.g. NSCLC not otherwise specified) were excluded from our analysis. In terms of definite tumor staging, pathological exploration was carried out post-surgically. Primary pulmonary lesions were pathologically classified based on the WHO 2004 guidelines; 149 specimens were classified as squamous cell carcinoma, 125 as adenocarcinoma and 47 as large cell carcinoma. Regular follow-up was performed for all patients, including systemic re-staging after 3, 6, 12, 18, 24, 36, 48 etc. months or earlier, if clinically required. Survival time was either computed from the date of histological diagnosis to death or to the date of last contact. Baseline information of the NSCLC population is shown in **Table 1**.

**Table 1:** Baseline characteristics of the study population (n = 321).

Parameter		n	% of non-missing values
**Sex**	Male sex	251	78
	Female sex	70	22
**Smoking status**	Non-smoker	61	20
	Smoker or ex-smoker	252	81
**Performance status**	ECOG 0	59	19
	ECOG I	235	76
	ECOG >II	16	5
**Adjuvant therapy**	Adjuvant chemotherapy	8	3
	Adjuvant radiotherapy	38	12
**Tumor stage**	Stage I	187	58
	Stage II	83	26
	Stage III	51	16
**Tumor size**	pT1	99	31
	pT2	192	60
	pT3	19	6
	pT4	11	3
**Lymph nodes status**	pN0	202	63
	pN1-3	117	37
**Tumor histology**	Squamous cell carcinoma	149	46
	Adenocarcinoma	125	39
	Large cell carcinoma	47	15
**Tumor grading**	G1	5	2
	G2	111	35
	G3-4	199	63
**Apoptosis**	Negative Bcl-2 expression	231	76
	Positive Bcl-2 expression	74	24
	Negative Bcl-xl expression	196	66
	Positive Bcl-xl expression	101	34
**EGFR mutation status**	No EGFR mutation	22	79
	EGFR mutation	6	21
**Proliferation (ki-67)**	Negative ki-67 expression	77	24
	Positive ki-67 expression	244	76
**PD-1 expression in tumor Infiltrating lymphocytes (TILs)**	negative lymphocytic expression	249	78
	negative lymphocytic expression	72	22
**PD-L1 expression in NSCLC**	negative tumor expression	244	76
	positive tumor expression	77	24

### Immunohistochemistry

Tissue microarrays were generated from formalin-fixed, paraffin-embedded tissue specimens (FFPE). In detail, three biopsy needle cores (core diameter at least 0.6 mm) of each tumor carefully selected to appropriately represent potential tumor heterogeneity were transferred to a recipient paraffin block as described [17]. For immunohistochemical analyses TMA slides were steam heated for 30 minutes in pH 6 citrate buffers, and subsequent immunostaining was performed with a 25 min incubation period of the primary antibody (DakoAutostainer, Denmark). The following primary monoclonal antibodies were applied: PD-1 (Abcam, ab 52587, mouse IgG1, clone NAT 105, 1:50), PD-L1 (Cell Signaling Technology, #13684 clone E1L3N, rabbit IgG1, 1:500), Bcl-2 (Santa Cruz Biotechnology, clone 100, mouse IgG1, 1:100) [18], Bcl-xl (Santa Cruz Biotechnology, clone H-5, epitope: C-terminus, mouse IgG1, 1:1000) [18], and ki-67 (Dako, M7240, clone MIB-1, mouse IgG1, 1:100). Immunoreaction was visualized with a biotinylated secondary antibody (LSAB/AP, #K5005 Dako) including the Red chromogen, according to the manufacturer. Finally, TMAs were counterstained with hematoxylin and covered with Cytoseal (Thermo Scientific, USA). Tonsillar tissue was employed as control for PD-1, PD-L1, Bcl-2 and ki-67 stainings, colon cancer tissue was used for the Bcl-xl. In accordance with previously published approaches in the field [19], the percentages of PD-1 positive lymphocytes and PD-L1 positive tumor cells were assessed using a semiquantitative score considering 0 as negative, 1 as weak, 2 as moderate and 3 as high. Tumors were evaluated as PD-L1 positive if ≥ 5% of the tumor cells displayed at least moderate staining. The tumor was evaluated as PD-1 positive if ≥ 5% of the lymphocytes displayed PD-1 staining. As described in Schmidt et al., Bcl-2 [18], Bcl-xl [18] and ki-67 were evaluated according to Remmele’s Immunoreactive Score (IRS range, 1–12, [20]). Here, cases were considered as positive if IRS was greater than or equal to 3. Analysis of TMA slides was performed by at least two independent investigators (L.H.S, T.S. and W.H.).

### EGFR analysis

The full protocol for EGFR analysis was previously published by Schmidt et al. [18]. In brief, DNA was extracted from FFPE tumor tissues and analyzed for EGFR mutations by Sanger sequencing. The EGFR status of each patient’s tumor was assessed from the individual status of all mutation types and recorded as one of the following: positive (mutation detected for at least one of the mutation types assayed), negative (no mutation detected in any of the mutation types assayed), or undetermined/ unknown (a positive or negative result could not be determined as per laboratory assessment).

### Statistical Analysis

The study population was described by standard descriptive statistical measures. For categorical variables, absolute and relative frequencies are reported. For continuous variables median and interquartile range (IQR) are reported, respectively. Association of clinico-pathological parameters with PD-1 and PD-L1 expression was tested using two-sided Fisher’s exact test. Univariate overall survival analysis was performed using the Kaplan-Meier method and log rank tests. A multivariable Cox proportional hazards model was fitted using a forward step-wise variable selection (inclusion criteria: p-value of the likelihood ratio test ≤0.05) to identify independent prognostic factors for overall survival. We considered potential prognostic factors that are tolerably complete (less than ten missing values, and with at least ten cases), to prevent statistical problems emerging from low sample size and extreme values. Patients with missing values in the cofactors were excluded from the analysis. All statistical tests were performed as exploratory analyses on a local significance level of 0.05. Since multiplicity adjustment was not carried out, no distinct overall significance level was ascertained. Hence, our findings may be used to set up new hypotheses. SPSS (SPSS Statistics, Version 22.0 released 2013, IBM Corp., Armonk, NY) was used for all statistical analyses.

## Results

### Immunohistochemistry

**Fig 1:**
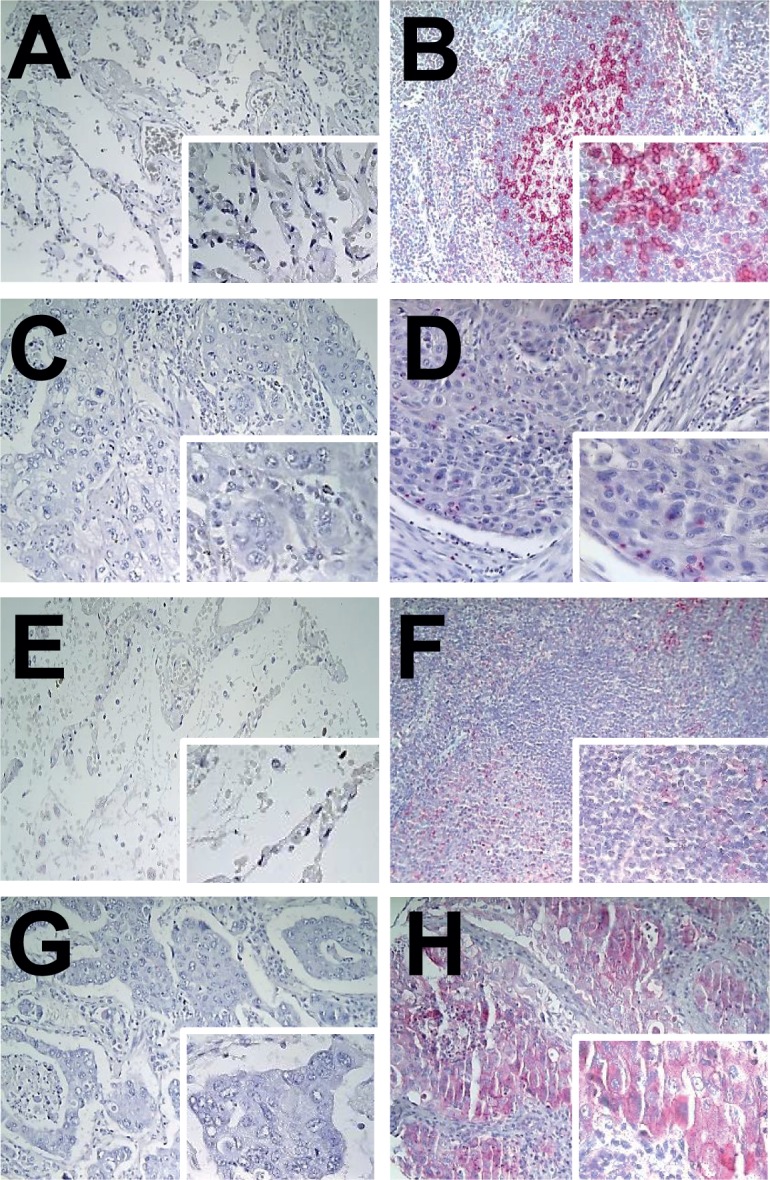
Representative immunohistochemical staining results for PD-1 (A: normal lung tissue, negative control; B: tonsillar tissue, positive control; C: PD-1-negative tumor infiltrating lymphocytes; D: PD-1-positive tumor infiltrating lymphocytes in squamous cell carcinomas) and for PD-L1 (E: normal lung tissue, negative control; F: tonsillar tissue, positive control; G: PD-L1 negative squamous cell carcinomas; H: PD-L1 positive squamous cell carcinomas). All images at x20, inlay x40.

The characteristics of the 321 NSCLC patients are summarized in **Table 1**. Due to tissue loss immunohistochemical evaluation was not feasible in 16 cases for Bcl-2 and in 24 cases for Bcl-xl. Positive PD-1 protein expression was found in 72 cases (22%) in tumor infiltrating lymphocytes (TILs). **Fig 1** demonstrates representative immunohistochemical staining patterns for normal lung tissue (**Fig 1A**), for control tonsillar tissue (**Fig 1B**) and for NSCLC (**Fig 1C and 1D**). PD-L1 was expressed by 24% of the NSCLC samples. Representative immunohistochemical staining patterns are given in **Fig 1E–1H** (**Fig 1E**: normal lung tissue, **Fig 1F**: control tonsillar tissue, **Fig 1G and 1H**: NSCLC). Tumor cells displayed a cytoplasmic staining pattern for PD-L1 (**Fig 1H**). Tumors with a PD-1 positive lymphocytic infiltrate displayed synchronous PD-L1 expression in 17 cases (5%). In case of heterogeneity with regard to the infiltrating PD-1 positive TIL population or PD-L1 positive tumor cells the fraction of positive lymphocytes/tumor cells was referred to the whole tumor tissue as represented by the selected tissue cores. Of interest, PD-1 positive TILs were rarely observed within the epithelial tumor cell formations, but rather within the tumor stroma, independent from PD-L1 expression status. Beyond PD-1 and PD-L1, immunohistochemical information for Bcl-2, Bcl-xl, ki-67 and EGFR mutation were available for the study collective [18]: 74 tumors (24%) involved in the study collective expressed Bcl-2, 101 tumors (34%) expressed Bcl-xl and EGFR mutations (positive, either at position 18, 19 or 21) were found for 6 patients (21%; **Table 1**). Of interest 16 samples (5%) concomitantly had PD1 expressing TILs and displayed PDL1 in the tumor cells.

### Clinicopathologic correlations

A positive correlation for PD-L1 tumor expression in NSCLC cells was found with Bcl-xl expression (p = 0.013). While 18% of Bcl-xl negative tissue samples expressed PD-L1, expression rate was 32% in Bcl-xl positive NSCLC samples. Besides this observed correlation, we did not find any other association for any of the other tested parameters (all p>0.05; **Table 2**).

**Table 2:** Associations of clinicopathological variables with PD-1 (in TILs) or PD-L1 (in NSCLC cells).

Variables		PD-1 (+) in TILs p-value* n (%)	PD-L1 (+) in NSCLC p-value* n (%)
**Age**	p-value	0.887	0.580
	<70 years	47 (22%)	49 (23%)
	≥70 years	25 (23%)	28 (26%)
**Sex**	p-value	0.260	0.637
	Male sex	60 (24%)	62 (25%)
	Female sex	12 (17%)	15 (21%)
**Smoking status**	p-value	0.384	0.315
	Non-smoker	10 (16%)	18 (30%)
	Smoker or ex-smoker	57 (23%)	57 (23%)
**Performance status**	p-value	0.120	0.867
	ECOG 0	18 (31%)	15 (25%)
	ECOG ≥1	52 (21%)	61 (24%)
**Adjuvant therapy**	p-value	0.849	0.712
	No adjuvant therapy	61 (22%)	65 (24%)
	Adjuvant therapy	11 (24%)	12 (26%)
**Tumor stage**	p-value	0.498	0.508
	Stage I	39 (21%)	42 (23%)
	Stage ≥1I	33 (25%)	35 (26%)
**Tumor size**	p-value	0.664	0.481
	pT1	24 (24%)	21 (21%)
	pT2-4	48 (22%)	56 (25%)
**Lymph nodes status**	p-value	0.489	0.892
	pN0	43 (21%)	48 (24%)
	pN1-3	29 (25%)	29 (25%)
**Tumor histology**	p-value	0.505	0.089
	Squamous cell carcinoma	36 (21%)	48 (28%)
	Non squamous cell carcinoma	36 (24%)	29 (20%)
**Tumor grading**	p-value	1.000	0.786
	< G2	25 (22%)	29 (25%)
	≥ G2	44 (22%)	47 (24%)
**Apoptosis**	p-value	0.523	0.162
	Negative Bcl-2 expression	50 (22%)	52 (23%)
	Positive Bcl-2 expression	19 (26%)	23 (31%)
	p-value	0.464	0.013
	Negative Bcl-xl expression	41 (21%)	36 (18%)
	Positive Bcl-xl expression	25 (25%)	32 (32%)
**EGFR mutation status**	p-value	0.288	1.000
	No EGFR mutation	7 (32%)	8 (36%)
	EGFR mutation	0	2 (33%)
**Proliferation (ki-67)**	p-value	0.756	0.287
	Negative ki-67 expression	16 (21%)	22 (29%)
	Positive ki-67 expression	56 (23%)	55 (23%)
**PD-1 expression in tumor Infiltrating lymphocytes (TILs)**	p-value		1.000
	negative lymphocytic expression		60 (24%)
	negative lymphocytic expression		17 (24%)
**PD-L1 expression in NSCLC**	p-value	1.000	
	negative tumor expression	55 (23%)	
	positive tumor expression	17 (22%)	

*p values according to Fisher’s exact test.

### PD-L1 expression indicates improved prognosis in NSCLC subgroups

**Fig 2:**
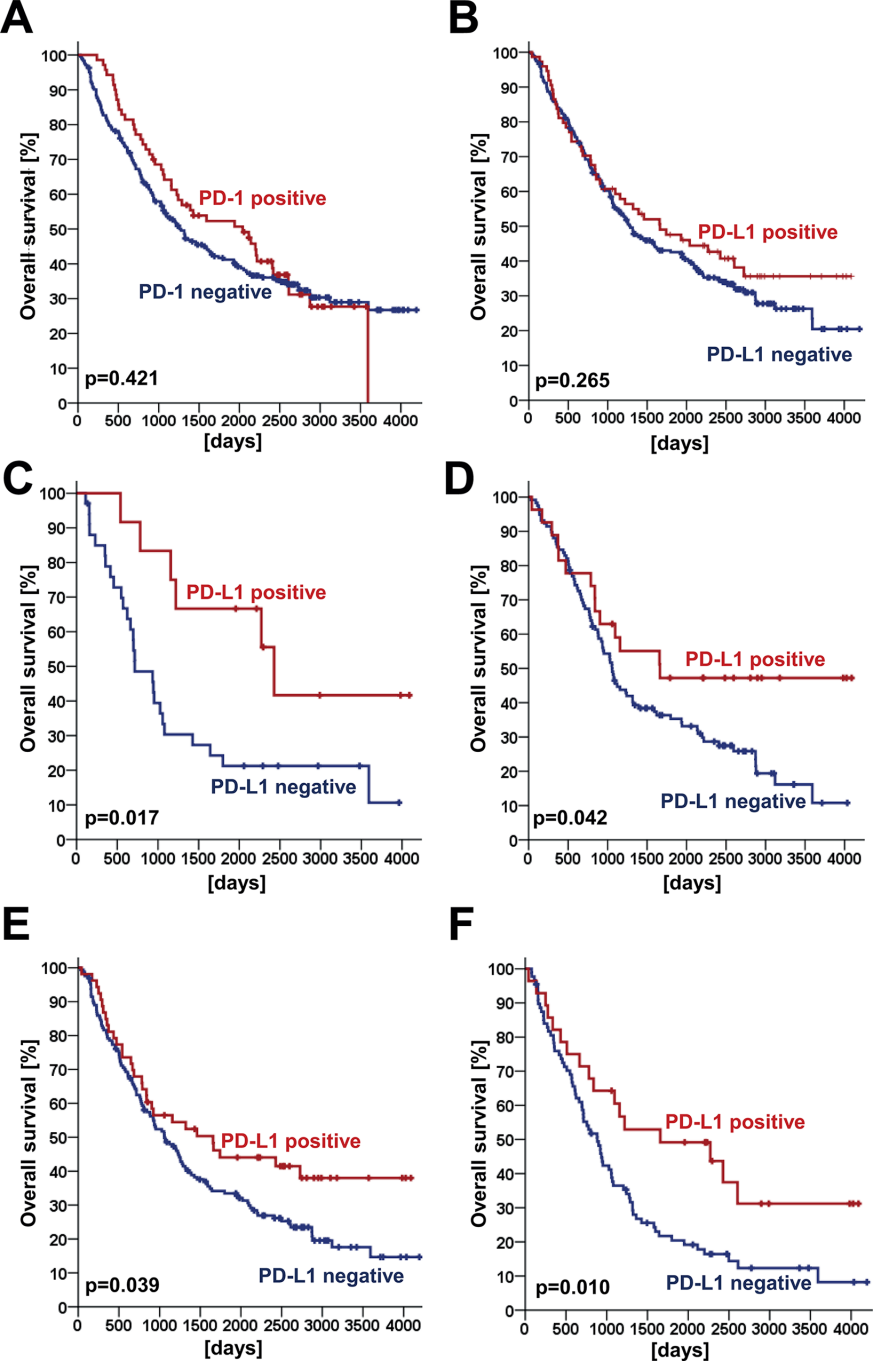
Prognostic impact of PD-L1 expression by tumor cells depends on tumor histology. Whereas for the full study collective (n = 321 patients), no prognostic effect was found, neither for PD-1 (A) nor for PD-L1 (B), patients who received adjuvant therapy (C), patients with pulmonary squamous cell carcinomas (D), patients with pT2-T4 tumors (E) and patients with a positive lymph node status (pN1-3, F) had an increased overall survival.

For the tested factors PD-1 and PD-L1 univariate Kaplan–Meier estimates for the full study collective did not demonstrate any significant effect on overall survival (OS). The p-values of the Log rank test are displayed for both, PD-1 (in TILs) and PDL1 (in NSCLC tumor cells) in **Table 3** and in **Fig 2** (**Fig 2A**: p-value for PD-1 in TILs = 0.421; **Fig 2B**: p-value for PD-L1 in NSCLC tumor cells = 0.265). Likewise concomitant expression of both factors did not show any relevant prognostic effect in the entire study cohort (p = 0.322; **S1 Fig**) as well as in subgroup analysis regarding all other tested parameters (data not shown).

**Table 3:** Univariate Log-rank test results for the association of PD-1 (in TILs) or PD-L1 (in NSCLC cells) with overall survival for defined subgroups.

Subgroups		PD-1 (+) in TILs p-value*	PD-L1 (+) in NSCLC p-value*
**All**		0.421	0.265
**Age**	<70 years	0.7790.054	0.2040.768
	≥70 years	0.7790.054	0.2040.768
**Sex**	Male	0.7640.125	0.0820.300
	Female	0.7640.125	0.0820.300
**Smoking status**	No smoking history	0.444	0.626
	Smoking history	0.324	0.358
**Performance status**	ECOG 0	0.467	0.150
	ECOG >I	0.749	0.780
**Adjuvant therapy**	No adjuvant therapy	0.255	0.797
	Adjuvant therapy	0.632	0.017
**Tumor stage**	Stage I	0.237	0.967
	Stage II-IV	0.807	0.052
**Tumor size**	pT1	0.893	0.303
	pT2-4	0.405	0.039
**Lymph node status**	pN0	0.274	0.644
	pN1-3	0.727	0.010
**Tumor histology**	Non squamous cell carcinoma	0.108	0.685
	Squamous cell carcinoma	0.619	0.042
**Tumor grading**	< G2	0.763	0.226
	≥ G2	0.249	0.663
**Bcl-2 expression**	Negative Bcl-2 expression	0.226	0.440
	Positive Bcl-2 expression	0.553	0.290
**Bcl-xl expression**	Negative Bcl-xl expression	0.437	0.466
	Positive Bcl-xl expression	0.475	0.712
**EGFR mutation status**	No EGFR mutation	0.018	0.597
	EGFR mutation		0.654
**Proliferation (ki67)**	Negative ki-67 expression	0.908	0.607
	Positive ki-67 expression	0.273	0.142
**PD-1 expression in TILs**	Negative PD-1 expression		0.132
	Positive PD-1 expression		0.465
**PD-L1 expression in NSCLC**	Negative PD-L1 expression	0.194	
	Positive PD-L1 expression	0.579	

*p values according to log rank test.

Subgroup analyses were performed for sex, age, smoking status, performance status, adjuvant therapy, tumor histology, tumor grading, tumor stage, tumor size, lymph node status, Bcl-2 expression, Bcl-xl expression, EGFR mutation status, PD-1 expression in tumor infiltrating lymphocytes and PD-L1 expression in tumor cells. Here, stratified analysis identified PD-L1 expression in NSCLC tumor cells to be associated with improved prognosis for adjuvant therapy (p = 0.017; **Fig 2C**), tumor histology (pulmonary squamous cell carcinoma; p = 0.042; **Fig 2D**), increased tumor size (pT2-4; p = 0.039; **Fig 2E**) and lymph node status (pN1-3; p = 0.010; **Fig 2F**). A prognostic effect was found for PD-1-positive tumor infiltrating lymphocytes in patients with non EGFR-mutated tumors (p = 0.018; **Table 3**). Due to the small number of patients (n = 22 patients), this effect is most likely not of clinical significance. Apart from this observation, further univariate subgroup analyses for PD-1 did not reveal any other relevant prognostic effect (all p>0.05; **Table 3**).

### Prognostic value of PD-1 and PD-L1

To determine the prognostic value of tumor infiltration by PD-1 positive lymphocytes and PD-L1 expression by tumor cells, Cox proportional hazards models for comparison with established prognostic factors were applied. As shown for the full study collective neither PD-1 nor PD-L1 were of prognostic relevance (all p>0.05, **Table 4**). Here only age (HR (95%CI) = 1.545 (1.147–2.080); p = 0.005), tumor stage (HR (95%CI) = 1.986 (1.492–2.645); p<0.001) and sex (HR (95%CI) = 1.658 (1.132–2.429; p = 0.006) were identified as prognostic parameters.

**Table 4:** Overall survival: Explanatory prognostic factors in a Cox proportional Hazards model for the full study collective and for subgroups depending on adjuvant therapy, tumor histology, tumor size and lymph node status. Included variables: PD-1 expression in tumor infiltrating lymphocytes (negative expression (ref.) *vs*. positive expression), PD-L1 expression in NSCLC cells (negative expression (ref.) *vs*. positive expression), sex (male (ref.) *vs*. female), age (<70 years (ref.) *vs*. ≥70 years), smoking status (no smoking history (ref.) *vs*. smoking history), adjuvant therapy (no adjuvant therapy (ref.) *vs*. adjuvant therapy), tumor histology (squamous cell carcinoma (ref.) *vs*. non squamous cell carcinoma), tumor stage (stage I (ref.) *vs*. stage II-IV), tumor size (pT1 (ref.) *vs*. ≥pT2), lymph node status (pN0 (ref.) *vs*. pN1-3) and grading (<G2 (ref.) *vs*. ≥G2).

Prognostic groups		Prognostic factor	p-value	HR^1^(95% CI)^2^
**All NSCLC patients (n = 301)**		Age	0.005	1.545 (1.147–2.080)
		Tumor stage	<0.001	1.986 (1.492–2.645)
		Sex	0.006	1.658 (1.132–2.429)
**Adjuvant therapy**	**No adjuvant therapy (n = 255)**	PD-1	0.035	0.659 (0.440–0.987)
		Age	0.019	1.471 (1.070–2.021)
		Tumor size	0.016	1.563 (1.074–2.275)
		Tumor stage	<0.001	1.928 (1.382–2.690)
	**Adjuvant therapy (n = 46)**	PD-L1	0.012	0.353 (0.145–0.861)
**Tumor histology**	**Non squamous cell carcinoma (n = 163)**	PD-1	0.030	0.561 (0.322–0.977)
		Age	0.016	1.704 (1.118–2.598)
		Sex	0.032	1.663 (1.028–2.688)
		Tumor size	0.043	1.627 (1.001–2.646)
		Lymph node status	0.001	2.049 (1.351–3.107)
	**Squamous cell carcinoma (n = 138)**	PD-L1	0.005	0.459 (0.252–0.833)
		Tumor stage	0.002	1.929 (1.277–2.913)
**Tumor size**	**pT1 (n = 93)**	Sex	0.002	2.860 (1.361–6.010)
		Tumor stage	0.005	2.615 (1.389–4.922)
	**pT2-4 (n = 208)**	PD-L1	0.004	0.556 (0.366–0.844)
		PD-1	0.023	0.626 (0.410–0.954)
		Age	0.041	1.429 (1.020–2.001)
		Lymph node status	0.001	1.783 (1.279–2.486)
**Lymph node status**	**pN0 (n = 192)**	Age	0.009	1.671 (1.140–2.449)
		Tumor size	0.004	1.809 (1.191–2.747)
	**pN1-3 (n = 109)**	PD-L1	0.005	0.470 (0.268–0.825)

Subgroup analyses were performed to prove the observed subgroup-relevant effects for PD-L1 with adjuvant therapy, tumor histology, tumor size and lymph node status. Cox regression models confirmed the observed positive prognostic effect of PD-L1 expression for adjuvant therapy (HR (95%CI) = 0.353 (0.145–0.861); p = 0.012; **Table 4**), for tumor histology (HR (95%CI) = 0.459 (0.252–0.833); p = 0.005; **Table 4**), for tumor size (HR (95%CI) = 0.556 (0.366–0.844); p = 0.004; **Table 4**) and for lymph node status (HR (95%CI) = 0.470 (0.268–0.825); p = 0.005; **Table 4**). For NSCLC patients who were not treated with adjuvant therapy (S2A Fig), patients with non-squamous cell carcinomas (**S2B Fig**), patients with small tumor sizes (pT1) or patients without lymphatic spread (pN0) no relevant prognostic effects were not found.

## Discussion

Blockade of inhibitory immune checkpoints is currently arising as a potential immunological option for tumor therapy. Targeting the PD-1/PD-L1 pathway in lung cancer has shown promise to positively affect prognosis in first clinical studies [13,21]. A more detailed understanding of the significance of the PD-1/PD-L1 pathway in this cancer is important to advance this promising treatment modality to its full potential. In order to evaluate the prognostic impact of tumor infiltration by PD-1 positive lymphocytes and PD-L1 expression by tumor cells, we performed a systematic study in a well-defined collective of 321 NSCLC patients undergoing primary tumor resection without preceding neoadjuvant therapy. Only completely resected, non-metastatic patients with a clear NSCLC histology were included for the statistical evaluation.

Tumor infiltration by PD-1 positive lymphocytes was detected in 22% of the tumor samples and 24% of the tumors displayed positivity for PD-L1 with 16 samples (5%) showing synchronous positivity for both. The finding concerning PD-L1 corresponds well to other published studies, which report immunohistochemical expression rates of 25–65% for PD-L1 in tumors of NSCLC patients [16,22–26]. Differences might be due to variabilities of the tumor microenvironment and to non-static expression at a single point of time [27]. To our knowledge, in contrast to reports on PD-L1 expression in tumor cells, no comparable data regarding the infiltration levels with PD-1 positive cells have been published, yet. Moreover, no mechanistic relation between the extent of PD-1 positive lymphocytic infiltration and PD-L1 positivity of the tumor cells has been shown so far. Hence, we did not find any association between PD-1 positive TILs and PD-L1 positive tumor cells.

To test whether PD-1 and PD-L1 correlate with the activation of apoptotic pathways [8], we analyzed associations for Bcl-2 and Bcl-xl. Here, positive associations were found for Bcl-xl expression in the tumor cells and PD-L1 expression in tumor cells. However, the observed positive correlation for Bcl-xl (Fisher’s exact test; **Table 2**) was not found in the prognostic subgroup analysis (log rank test; **Table 3**). So far, the impact of this observation is not clear and it may not be relevant. While one study group reported PD-L1 expression to be associated with adenocarcinoma histology [15] and another study reported it to be associated with squamous cell carcinoma histology [14], our correlation anaylsis (Fisher’s exact test) supports the latter observation. As shown before, we did not find any association between PD-L1 expression and presence of EGFR mutations [26].

The prognostic analysis of the full study cohort, including all NSCLC histologies, did not reveal any significant effect of tumor infiltration by PD-1 positive lymphocytes and/or PD-L1 expression of the tumor cells on overall survival (**Fig 2A and 2B**). However, a favorable prognosis was found for PD-L1 expression in tumor cells for patients who received adjuvant therapy, with pulmonary squamous cell carcinomas, higher T descriptor or lymph node metastasis (**Fig 2C and 2D**). Beyond other prognostic variables such as age or tumor stage, multivariate analyses confirmed PD-L1 expression in tumor cells to be a marker for an improved prognosis for patients with these characteristics. For infiltration by PD1 positive lymphocytes borderline prognostic effects were found in some multivariate subgroup analyses.

With respect to the recent literature, previous studies regarding the prognostic role of PD-L1 for NSCLC patients have been controversial. There are studies suggesting a negative prognostic value [15,24], whereas others did not find any prognostic impact [22,24,26]. Recently, one larger study including 340 NSCLC patients reported both, tumor PD-L1 protein and PD-L1 mRNA expression to be associated with increased local lymphocytic infiltrates and increased overall survival [14]. Our results are in agreement with this and further previous reports in other tumor types. An association between PD-L1 expression with an improved overall survival was found in metastasized malignant melanoma [28], colorectal cancer [29], and breast cancer [30]. As in our study, all reported patients did not receive anti-PD1/PD-L1 therapies.

The biology of an association between PD-L1 expression and better outcome in patients with adjuvant therapy, lymphatic metastasis, and squamous cell carcinoma is not well understood. A potential explanation is that the favorable prognostic impact of PD-L1 upregulation in these conditions may indicate the presence of a mixed immune cell infiltrate containing cytotoxic and regulatory T cells and reflect a partially dysbalanced local cellular immune response, which still contributes to antitumor immune control. In this case, a specific therapeutic interference with the PD-1/PD-L1 pathway may unleash a cytotoxic T cell response in the tumor. Thus, more detailed studies on the phenotype of infiltrating immune cells, in particular with regard to T cell subpopulations, seem to be important. Of interest, neither the proliferation marker Ki-67 nor the anti-apoptotic factors were associated with the prognostic effect of PD-L1 expression, highlighting that regulation of proliferation and apoptosis may be independent form immunologic mechanisms.

With respect to therapeutic interventions, inhibition of PD-1/PD-L1 is expected to become a powerful therapeutic alternative for NSCLC [31]. Overall, for advanced NSCLC patients, the overall response rate (ORR) for PD-1 inhibitory drugs was 24%, whereas for NSCLC patients with PD-L1 expression the ORR was 100% compared to 15% for PD-L1 negative tumors [32]. The latter study argues in favour of the evaluation of PD-L1 expression as a selective biomarker, and the analysis of PD-L1 in NSCLC could serve as predictor for response to PD-1 pathway inhibition and additionally as a prognostic marker for improved clinical outcome [33].

Our oligocentric study has several limitations, such as its retrospective nature and the potential risk of bias resulting from variable treatment protocols, regarding both surgical procedures and adjuvant therapies. The issue of representative tissue sample selection for TMAs was addressed in a previous study [18]. To reduce sampling errors, each patient’s tumor was represented by three tissue cores sampled from different tumor areas covering potential histological heterogeneity.

In conclusion, PD-L1 is a prognostic factor for NSCLC patients with squamous cell carcinoma histology, lymph node metastasis and patients treated in an adjuvant setting. It is feasible to hypothesize that patients with PD-L1 expression profit the most in an adjuvant treatment setting, however the sample size of our study is too small to answer this clinically important issue. Prospective studies are required to confirm this observation. If our observation is confirmed by further and prospective analyses, PD-L1 expression could contribute to adequate risk stratification. Beyond conventional therapies, PD-L1 expression likely represents a critical biomarker for predicting the individual probability of response to treatment with PD-1/PD-L1 pathway inhibitory agents. Prospective assessment of this parameter along with clinical trials will help to establish its significance in this context and allow selecting patients with a high likelihood to respond to various therapeutic interventions.

## Supporting Information

S1 Fig Prognostic impact of PD-1, PD-L1 and simultaneous PD-1/PD-L1 expression in the total study cohort. (EPS)

S2 Fig Prognostic impact of PD-L1 in patients who did not receive any adjuvant treatment (A) and NSCLC patients with non-squamous cell carcinomas (B). (EPS)
